# Salidroside Decreases Atherosclerotic Plaque Formation in Low-Density Lipoprotein Receptor-Deficient Mice

**DOI:** 10.1155/2012/607508

**Published:** 2012-10-10

**Authors:** Bu-Chun Zhang, Wei-Ming Li, Rong Guo, Ya-Wei Xu

**Affiliations:** Department of Cardiology, Shanghai Tenth People's Hospital, Tongji University School of Medicine, 301 Yanchang Road, Shanghai 200072, China

## Abstract

Salidroside is isolated from *Rhodiola rosea* and is one of the main active components in *Rhodiola* species. The present study was designed to evaluate the effects of Salidroside on atherosclerotic plaque formation in high-fat diet-(HFD-) fed female LDL receptor knockout (LDLr^−/−^) mice. LDLr^−/−^ mice fed an atherogenic HFD for 12 weeks were divided into two groups. One group was administered Salidroside (50 mg/kg/oral gavage) daily for 8 weeks, while the control group was administered saline. Salidroside treatment reduced serum lipids levels and the plaque area through the arch to the abdominal aorta. Furthermore, Salidroside improved macrophage content and enhanced collagen and smooth muscle cells contents in the aortic sinus. These changes were associated with reduced MCP-1, VCAM-1, and VCAM-1 protein expression in atherosclerotic aortas. All these results suggest that Salidroside decreases atherosclerotic plaques formation via effects on lipid lowering and anti-inflammation in HFD-fed LDLr^−/−^ mice.

## 1. Introduction

Atherosclerosis (AS) is a widespread and one of the most dangerous cardiovascular diseases which cause considerable threat to human health worldwide. Accordingly, treatment aimed at AS is of great clinical importance. However, an ideal drug against atherosclerosis is still lacking [1]. In China, drugs of herbal origin with low side effects are of high interest as alternative therapy, and medicinal plants may have potential to stabilize atherosclerotic plaques [2].

Rhodiola rosea has long been used as a medicinal plant and has been reported to have various pharmacological properties, including antifatigue and antistress activity [3], anticancer, antioxidant and immune enhancing and stimulating sexual activity [4], anti-inflammation [5], improvement of glucose and lipid metabolism [6, 7], antiarrhythmic effect [8], and enhancement of angiogenesis [9]. However, the effects of Rhodiola rosea on atherosclerotic lesions formation are still unclear. The aim of the present study was to evaluate the effects of Salidroside (p-hydroxyphenethyl-*β*-d-glucoside, one of the main active components in Rhodiola species) on atherosclerotic plaque formation in high-fat diet-(HFD) fed female LDL receptor knockout (LDLr^−/−^) mice.

## 2. Materials and Methods

### 2.1. Animal Model

Female LDLr^−/−^ mice (C57BL/6 genetic background) were obtained from the Jackson Laboratory (Bar Harbor, ME, USA). A total of 30 LDLr^−/−^ mice (3 weeks old, weight 19 to 21 g) were used in the study. All animal procedures were performed in compliance with “The Guide for the Care of Use of Laboratory Animals” published by the National Institute of Health (NIH Publication No. 85-23, revised 1996) and approved by the Animal Care Committee of Tongji University School of Medicine. Mice were fed a high-fat diet (containing 18% hydrogenated cocoa butter, 0.15% cholesterol, 7% casein, 7% sucrose, and 3% maltodextrin) for 12 weeks, starting at 4 weeks of age. Thirty mice were further divided into two groups randomly (*N* = 15 per group). One group received Salidroside (≥98% purity, purchased from Yuanye Technology Co., Ltd., Shanghai, China) 50 mg/kg/oral gavage once daily. The Salidroside dose used was selected from a pilot experiment, in which we aimed at only 20% cholesterol reduction to ensure atherosclerosis development within a reasonable time period. while the other group received saline. During the 8 weeks of treatment, all mice allowed free access to a high-fat diet and water.

### 2.2. Serum Lipid Analysis

Blood samples were taken after a 4-hour fast by tail bleeding throughout the study. Serums were acquired through centrifugation of the blood samples at 4°C at 1000 g and stored at −80°C until analysis. Total cholesterol (TC), high-density lipoprotein cholesterol (HDL-C), and triglyceride (TG) levels were measured enzymatically with commercial kits from Wako Inc. (Richmond, USA) using an auto-analyzer (Hitachi 7100, Tokyo, Japan).

### 2.3. Morphology of Atherosclerotic Plaques

After 8 weeks of treatment, mice were anesthetized by diethyl ether and sacrificed. The mice were dissected and aortas were perfusion-fixed with 4.5% formaldehyde. Then the aortas were dissected from the heart to approximately 3 mm distal to the iliac bifurcation. The aortas were preserved in fresh paraformaldehyde solution for 2 weeks, and oil red O staining was employed to determine the plaques on entire aortas. Briefly, after removing surrounding adventitial fatty tissue, the aortas were opened longitudinally and pinned out on a black silica gel plate. The aorta was rinsed in 70% ethanol after 12 hours of fixation in the paraformaldehyde solution, stained with 0.05% oil red O solution in 50% acetone/35% ethanol for about 10 minutes, and washed in 80% ethanol for 5 minutes. For collagen determination, picric and sirius staining was performed, according to the manufacturer's instructions (Genmed Inc., Arlington, USA). Finally, the stained aortas were photographed and analyzed using NIH Image Pro-Plus 6.0 software (NIH, Bethesda, MD).

### 2.4. Immunohistochemical Staining of *α*-SMA and MAC-3 in the Aortic Sinus

Isolated aortic sinus tissues were fixed by immersion in 4% paraformaldehyde for 48 h at 4°C and incubated with 30% sucrose for 2 days. Each aortic sinus was embedded in paraffin. The paraffin-embedded sections (5 um thick) were placed on poly-L-lysine-coated slides. The slides were air dried overnight at room temperature, wrapped, and stored at −70°C until immunostaining. Slides were immersed in 0.3% H_2_O_2_ for 10 minutes to abolish endogenous peroxidase activity and rinsed with PBS. And then, slides were incubated with 5% BSA for 1 hour at room temperature to block nonspecific staining and incubated with a primary antibodies of murine *α*-smooth muscle actin (*α*-SMA) antibody (1 : 50 dilution; Santa Cruz Inc., California, USA), MAC-3 (1 : 50 dilution; Beyotime Biotech Inc., Jiangsu, China) in humidified chambers for overnight at 4°C. All slides were incubated with biotinylated secondary antibody for 1 hour at room temperature and then incubated with horseradish peroxidase-conjugated streptavidin for 20 min at room temperature, followed by detection with a DAB kit (Beyotime Biotech Inc., Jiangsu, China). For the quantitative analysis, the average score of 10–20 randomly selected area was calculated using NIH Image Pro-Plus 6.0 software.

### 2.5. Western Blot Analysis

Aorta sinus tissues were snap-frozen in liquid nitrogen, pulverized, and resuspended in ice-cold lysis buffer (Solarbio). Protein concentrations were determined with the Bradford method. Lysates were allowed to solubilize on ice for 30 min, and particulate mass was removed by centrifugation (15,000 g) for 15 min at 4°C. Supernatants were analyzed by SDS-PAGE. Primary antibodies used included intercellular adhesion molecule 1 (ICAM-1, 1 : 1000 dilution), vascular cell adhesion molecule 1 (VCAM-1, 1 : 1000 dilution) and monocyte chemotactic protein-1 (MCP-1, 1 : 500 dilution) were purchased from Santa Cruz Inc. (California, USA). Secondary antibodies were horseradish peroxidase-labeled antibodies (Thermo Scientific Pierce, Rockford, USA). Blots were processed for enhanced chemifluorescence using a Pierce ECL Western blotting substrate (Thermo Scientific Pierce, Rockford, USA).

### 2.6. Statistical Analysis

All statistical analyses were carried out with GraphPad PRISM 5.0 statistical software (San Diego, California, USA). Quantitative variables are expressed as means ± SD. Two-tailed Student's *t*-tests were used to compare continuous data for between-group differences. *P* < 0.05 was considered statistically significant.

## 3. Results

### 3.1. Body Weights and Biochemical Studies

The weight of mice was 20.09 ± 0.40 g at baseline but increased to 31.68 ± 0.15 g at week 24 (*P* < 0.001). However, there was no significant difference in body weight among the two groups at either baseline or week 24. Compared with the vehicle group receiving no drugs for 8 weeks, mice treated with Salidroside showed lower levels of TC and TG and significantly increased HDL-C (Table 1). These findings suggest that Salidroside intervention may help to restore the lipid imbalance induced by HFD.

### 3.2. Salidroside Significantly Reduces the Formation of Atherosclerotic Lesions

**Figure 1:**
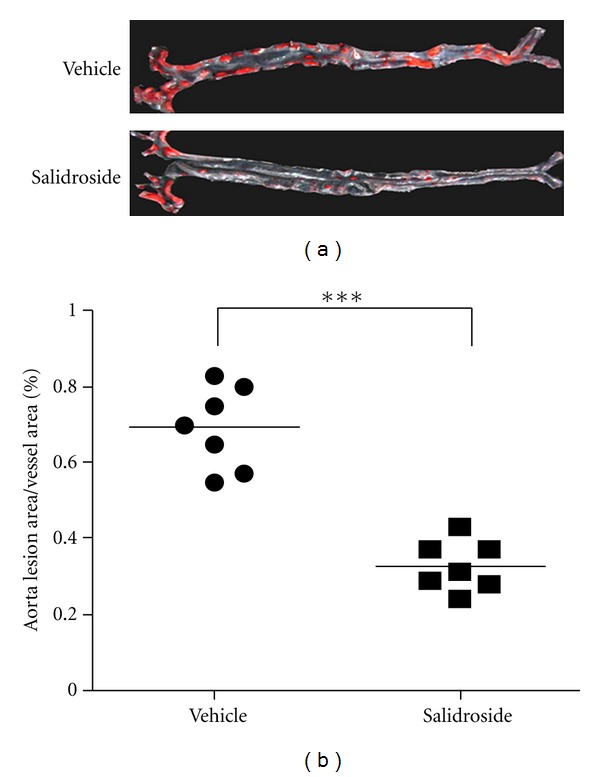
Salidroside decreases the atherosclerotic area. (a) Representative en face atherosclerotic aorta preparations stained with oil red O. (b) Comparison of plaque sizes between the vehicle and Salidroside groups (*N* = 7 per group). The area stained with dye is expressed as a percentage of the total surface area. The mean is depicted as a single horizontal line, ***P < 0.001 versus vehicle group.

To ascertain the effects of Salidroside on atherosclerotic lesion formation, we detected plaque sizes at the aorta via oil red O staining. As shown in Figure 1, Salidroside induced a significant decrease in the plaque area. Vehicle-treated mice group displayed approximately 69.29 ± 0.04% plaque coverage, whereas mice treated with Salidroside had 32.71 ± 0.02% plaque coverage.

**Figure 2:**
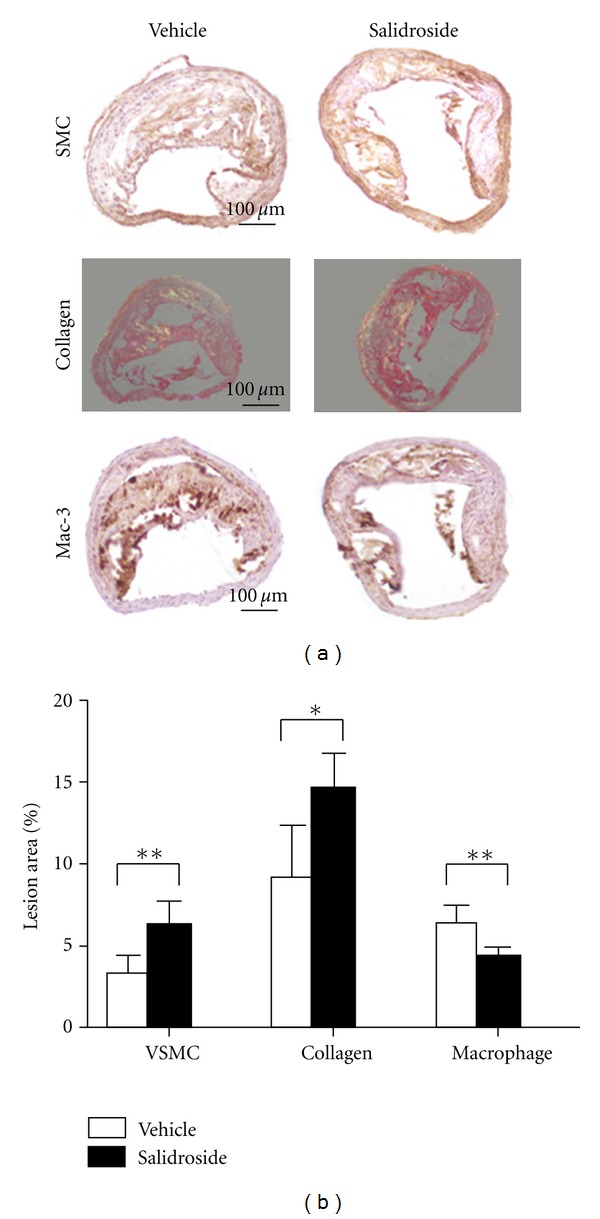
Composition of atherosclerotic plaques in the aortic sinus. (a) Representative examples are provided for VSMC-Actin, collagen regions (sirius red), and macrophage antibody (Mac-3). (b) Comparison of VSMC, macrophage, and collagen content between the two groups (*N* = 5 per group), *P < 0.05 and **P < 0.01 versus vehicle group.

To evaluate lesion composition, we immunostained the lesions for macrophages, vascular smooth muscle cells (VSMCs), as well as collagen content. The positive staining area of *α*-actin in the Salidroside-treated groups was significantly higher than that in the vehicle group (6.21 ± 1.48% versus 3.25 ± 1.17%, *P* < 0.01). Similarly, the area positively stained with sirius red in the Salidroside-treated groups was significantly higher than that in the vehicle group (14.51 ± 2.24% versus 9.12 ± 3.22%, *P* < 0.05); however, the area stained positive for MAC-3 was significantly lower than that in the vehicle group (4.27 ± 0.65% versus 6.32 ± 1.15%, *P* < 0.01) (Figure 2).

### 3.3. Salidroside Decreases ICAM-1, VCAM-1, and MCP-1 Expression in the Lesion Areas

**Figure 3:**
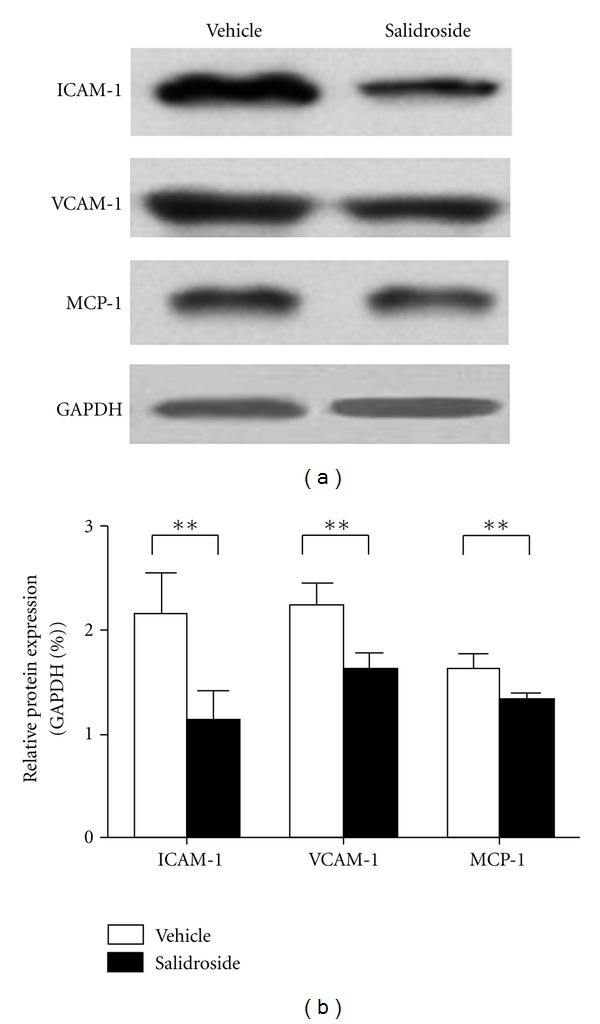
Salidroside attenuates inflammatory mediators expression at the aortic arch. (a) Representative western blotting for ICAM-1, VCAM-1, and MCP-1. (b) Quantitative analysis (*N* = 5 per group), **P < 0.01 versus vehicle group.

We additionally evaluated the expression of several inflammatory mediators in the aortic arch plaques using Western blot. As shown in Figure 3, the protein levels of ICAM-1, VCAM-1, and MCP-1 in the Salidroside-treated groups were significantly lower than that in the vehicle group (all *P* < 0.01).

## 4. Discussion

The aim of this study was to evaluate the effects of Rhodiola rosea on atherosclerosis development in high-fat diet-fed mice. Salidroside has been shown to reduce atherosclerostic plaque formation, serum levels of lipids, and vascular inflammatory markers. These results demonstrated for the first time that Rhodiola rosea provides a novel approach to against atherosclerosis.

Previous studies have demonstrated that atherosclerosis is a complex and perpetuating inflammatory disease involving the aorta and its major branches, and the distinctive histological features of vulnerable plaques in humans include a large lipid core, a thin fibrous cap depleted of extracellular matrix and VSMCs, active inflammation, outward or positive remodeling, and increased adventitial and plaque neovascularity [10]. In the present study, Salidroside treatment decreased protein levels of ICAM-1, VCAM-1, and MCP-1 in plaques. In addition, the macrophage content in the lesion area in the Salidroside-treated group was lower than that of the vehicle group. All of these inflammatory factors were previously shown to be involved in inflammatory cascade and have been implicated in the pathogenesis of atherosclerosis and plaque destabilization [11].

Another noteworthy finding is that the collagen and VSMCs contents in the lesion area in the Salidroside-treated group were higher than that of the vehicle group. Vascular remodeling, especially extracellular matrix (ECM) increase, is thought to stabilize plaques, which may prevent disruption of lesions [12]. Based on the present data, we propose that Salidroside increases the collagen content or decreases degradation of collagen in the lesion areas, indicating that Salidroside stabilizes plaques.

Our study contains several limitations. First, although components of Salidroside were clear, the dose-effect relationship of Salidroside on atherosclerotic plaques progression remains to be clarified. Second, although the antiatherosclerosis effects of Salidroside have been confirmed, the detailed molecular mechanism of the lipid-lowering and anti-inflammation effects requires further investigation.

In summary, our *in vivo* studies demonstrate that Salidroside decreases atherosclerotic plaques formation via effects on lipid lowering and anti-inflammation in high-fat diet-fed LDLr^−/−^ mice. Thus, treatment with Rhodiola rosea provides a new therapeutic approach to prevent atherosclerosis.

## Figures and Tables

**Table 1:** Serum lipid profile in two groups after 8-week treatment.

Groups (*n* = 15)	TC (mg/dL)	TG (mg/dL)	HDL-C (mg/dL)
Vehicle	1178.7 ± 123	463.6 ± 33	18.5 ± 2.1
Salidroside	524.3 ± 65***	237.5 ± 17***	20.7 ± 1.6**

TC: total cholesterol; TG: triglyceride; HDL-C: high-density lipoprotein.

***P < 0.001 **P < 0.01 versus vehicle group.

## References

[1] Libby P, Aikawa M (2002). Stabilization of atherosclerotic plaques: new mechanisms and clinical targets. *Nature Medicine*.

[2] Liu Y, Yan F, Liu Y (2008). Aqueous extract of rhubarb stabilizes vulnerable atherosclerotic plaques due to depression of inflammation and lipid accumulation. *Phytotherapy Research*.

[3] Darbinyan V, Kteyan A, Panossian A, Gabrielian E, Wikman G, Wagner H (2000). *Rhodiola rosea* in stress induced fatigue—a double blind cross-over study of a standardized extract SHR-5 with a repeated low-dose regimen on the mental performance of healthy physicians during night duty. *Phytomedicine*.

[4] Calcabrini C, De Bellis R, Mancini U (2010). *Rhodiola rosea* ability to enrich cellular antioxidant defences of cultured human keratinocytes. *Archives of Dermatological Research*.

[5] Bawa PAS, Khanum F (2009). Anti-inflammatory activity of *Rhodiola rosea*—'A second-generation adaptogen'. *Phytotherapy Research*.

[6] Gao D, Li Q, Liu Z (2009). Antidiabetic potential of Rhodiola sachalinensis root extract in streptozotocin-induced diabetic rats. *Methods and Findings in Experimental and Clinical Pharmacology*.

[7] Wang J, Rong X, Li W, Yang Y, Yamahara J, Li Y (2012). *Rhodiola crenulata* root ameliorates derangements of glucose and lipid metabolism in a rat model of the metabolic syndrome and type 2 diabetes. *Journal of Ethnopharmacology*.

[8] Maslov LN, Lishmanov YB (2007). Cardioprotective and antiarrhythmic properties of *Rhodiola rosea* preparations. *Eksperimental’naya i Klinicheskaya Farmakologiya*.

[9] Shen W, Fan WH, Shi HM (2008). Effects of rhodiola on expression of vascular endothelial cell growth factor and angiogenesis in aortic atherosclerotic plaque of rabbits. *Zhongguo Zhong Xi Yi Jie He Za Zhi*.

[10] van Diepen S, Roe MT, Lopes RD (2012). Baseline NT-proBNP and biomarkers of inflammation and necrosis in patients with ST-segment elevation myocardial infarction: insights from the APEX-AMI trial. *Journal of Thrombosis and Thrombolysis*.

[11] Stoll G, Bendszus M (2006). Inflammation and atherosclerosis: novel insights into plaque formation and destabilization. *Stroke*.

[12] Finn AV, Nakano M, Narula J, Kolodgie FD, Virmani R (2010). Concept of vulnerable/unstable plaque. *Arteriosclerosis, Thrombosis, and Vascular Biology*.

